# Construction and verification of machine vision algorithm model based on apple leaf disease images

**DOI:** 10.3389/fpls.2023.1246065

**Published:** 2023-09-13

**Authors:** Gao Ang, Ren Han, Song Yuepeng, Ren Longlong, Zhang Yue, Han Xiang

**Affiliations:** ^1^ College of Mechanical and Electronic Engineering, Shandong Agricultural University, Tai’an, Shandong, China; ^2^ Key Laboratory of Horticultural Machinery and Equipment of Shandong Province, Shandong Agricultural University, Tai’an, Shandong, China; ^3^ Intelligent Engineering Laboratory of Agricultural Equipment of Shandong Province, Shandong Agricultural University, Tai’an, Shandong, China

**Keywords:** apple leaf disease, deep learning, deep separable convolution, re-parameterization, leaf detection network

## Abstract

Apple leaf diseases without timely control will affect fruit quality and yield, intelligent detection of apple leaf diseases was especially important. So this paper mainly focuses on apple leaf disease detection problem, proposes a machine vision algorithm model for fast apple leaf disease detection called LALNet (High-speed apple leaf network). First, an efficient sacked module for apple leaf detection, known as EALD (efficient apple leaf detection stacking module), was designed by utilizing the multi-branch structure and depth-separable modules. In the backbone network of LALNet, (High-speed apple leaf network) four layers of EALD modules were superimposed and an SE(Squeeze-and-Excitation) module was added in the last layer of the model to improve the attention of the model to important features. A structural reparameterization technique was used to combine the outputs of two layers of deeply separable convolutions in branch during the inference phase to improve the model’s operational speed. The results show that in the test set, the detection accuracy of the model was 96.07%. The total precision was 95.79%, the total recall was 96.05%, the total F1 was 96.06%, the model size was 6.61 MB, and the detection speed of a single image was 6.68 ms. Therefore, the model ensures both high detection accuracy and fast execution speed, making it suitable for deployment on embedded devices. It supports precision spraying for the prevention and control of apple leaf disease.

## 1 Introduction

There are approximately more than 80 countries worldwide engaged in large-scale apple production, and as the area under apple production continues to expand (Zhang, 2021), the incidence of pests and diseases affecting apples has become increasingly severe. Apple leaf diseases, if left untreated, would pose a serious threat to the growth, development and quality of apples. Currently, traditional methods of diagnosing apple leaf diseases rely heavily on human judgment, requiring experienced and highly skilled field workers. Errors in worker judgment can lead to delayed prevention or excessive control measures, both of which can be detrimental. Therefore, efficient and rapid assessment of apple leaf diseases plays a critical role in improving apple quality and increasing grower profitability.

With the development of computer vision and artificial intelligence, deep learning has received increasing attention in the field of image processing (Shun et al., 2019; Zhang and Lu, 2021), while deep learning techniques have a wide range of ap plications in agriculture (Kamilaris and Prenafeta-Boldú, 2018; Zheng et al., 2019; Sharma et al., 2020; Arumugam et al., 2022). In the research of plant leaf disease classification, Aditya Karleka et al. designed a deep learning convolutional neural network Soybean leaf diseases classification (SoyNet) by increasing the diversity of pooling operations, adding Relu functions and dropout operations rationally for identifying and classifying soybean plant The proposed model achieved 98.14% recognition accuracy with good precision, recall and F1 score (Guo et al., 2022). Paul Shekonya Kanda et al. proposed an intelligent method based on deep learning to identify nine common tomato diseases. The method employed a residual neural network algorithm to identify tomato diseases and used five network depths to measure the accuracy of the network. According to the experimental result, this method obtained the highest F1 score of 99.5%, outperforming most previous competing methods in tomato leaf disease identification (Zhang et al., 2021). Laixiang Xu et al. proposed a new deep learning model for peanut leaf disease recognition. This proposed model was a combination of an improved X-ception, a partially activated feature fusion module and two attention enhancement branches. The model obtained 99.69% accuracy in the test set, which is 9.67% - 23.34% higher than Inception-V4, ResNet 34 and MobileNet-V3, demonstrating the feasibility of the model (Gill and Khehra, 2022). It shows that by designing specific network parameter settings in convolutional neural networks for plant disease classification, adding residual structure, adding attention mechanism, and other operations were capable of achieving higher accuracy.

In the study of apple leaf disease classification convolutional neural network model numerous scholars have done a lot of researches on improving the accuracy of apple tree leaf disease classification recognition, reducing the parameters and training time of specific recognition networks. For example, Yong et al. proposed a DenseNet-121 deep convolutional network based on three methods of regression, multi-label classification and focal loss function to identify apple leaf diseases. The proposed method achieved 93.51%, 93.31%, and 93.71% accuracy on the test set, respectively, outperforming the traditional cross-entropy loss function-based multi-classification method with 92.29% accuracy (Zhong and Zhao, 2020). Lili et al. proposed a convolutional neural network based on the AlexNet model for the classification of five diseases of apple tree leaves, which uses dilated convolution to extract coarse-grained features of diseases in the model, which helps to reduce the number of parameters while maintaining a large field of perception, and adds parallel convolutional modules to extract leaf disease features at multiple scales. Subsequently, a series of 3 × 3 convolutional shortcut connections allowed the model to handle additional nonlinearities. The final recognition accuracy of the model was 97.36% and the model size was 5.87 MB (Li et al., 2022). Qian et al. proposed an improved model based on VGG16 to identify apple leaf diseases, in which a global average polarization layer was used instead of a fully connected layer to reduce parameters and a batch normalization layer was added to improve convergence speed. A migration learning strategy is used to avoid long training time. The experimental results show that the overall accuracy of apple leaf classification based on the proposed model could reach 99.01%. Compared with the classical VGG16, the model parameters are reduced with 89%, the recognition accuracy is improved with 6.3%, and the training time is reduced to 0.56% of the original model (Yan et al., 2020).

In apple leaf disease classification and recognition research, scholars have achieved high recognition accuracy using deep learning techniques, however, how to ensure apple leaf disease recognition accuracy while making the model run faster is still the focus of research. Therefore, this paper proposes the LALNet model, in the next section in-depth discussion of the research content of this paper, in the second section, mainly introduces the data set of this paper, the main components of the LALNet network using the multi-branching structure and the depth separable module to design the efficient leaf detection EALD module, in the LALNet in the use of the EALD module stacking and add SE attention module, Finally, in the inference stage using structural re-parameterization technique to improve the running speed of the model. In Section III, the model was trained, validated and tested using publicly available apple leaf disease datasets, and a comparative analysis of this paper’s model with state-of-the-art apple leaf classification models was performed to provide a comprehensive evaluation of the model to ensure its reliability. In Sec. IV, the research work of this paper was fully summarized and the limitations of this research and future research directions were discussed. Thus, the proposed LALNet model improves the speed of image recognition while ensuring recognition accuracy, and finally, this research can support intelligent apple leaf spray control.

## 2 Tests and methods

### 2.1 Apple leaf data set

In this study, apple leaf disease images were collected at the apple experimental field of Shandong Agricultural University (117.12°E,36.20°N) and at the Tianping Lake experimental demonstration base of Shandong Fruit Tree Research Institute, National Apple Engineering Technology Research Center (117.01°E,36.21°N), which were collected several times in July 2022 under favorable weather conditions.

**Figure 1:**
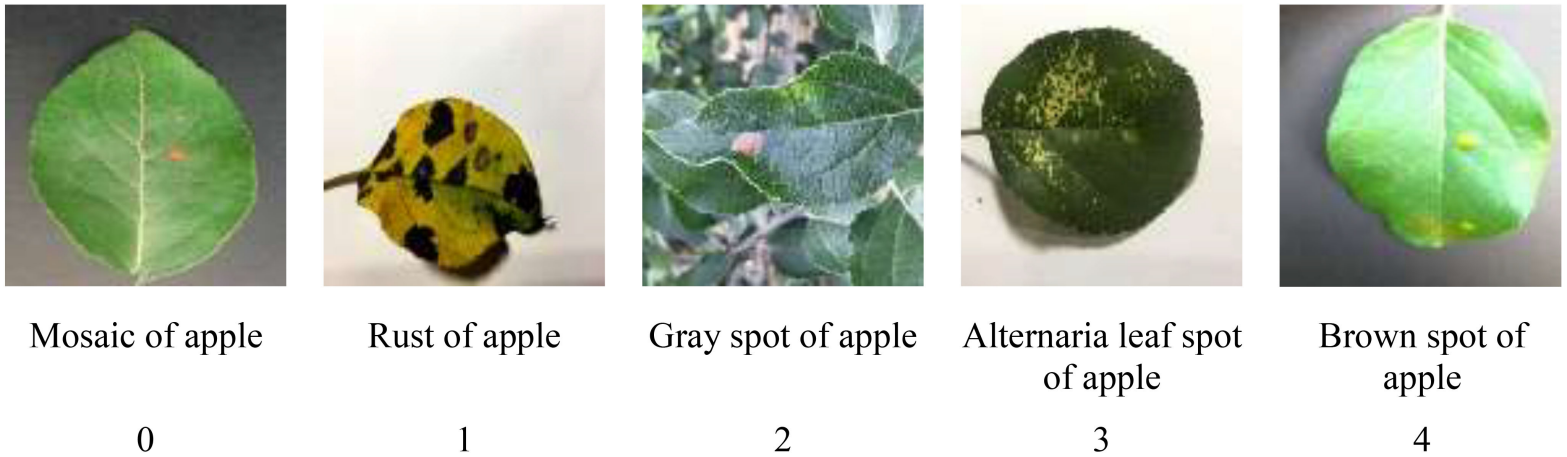
Pictures of some fruit leaf diseases.

Meanwhile, Baidu public dataset of apple leaf pathology images (Ai Studio poublic datasets, 2023) was used to expand the dataset of this paper. This dataset contains five types of common apple leaf diseases, namely apple mosaic, rust, gray spot, alternaria leaf spot and brown spot. For the convenience of training management, apple mosaic, rust, gray spot, alternaria leaf spot, and brown spot were represented by the numbers 0, 1, 2, 3, and 4, respectively, and some apple leaf disease images are shown in **Figure 1**. After flipping, panning and contrast enhancement to pre-process the data set of this paper, a total of 25,000 disease images with image size of 224*224 were obtained. In order to use this dataset for training, validation and testing, the data is divided as shown in **Table 1**, 80% of the images were used for model training, 10% of the images were used for model validation and 10% of the images were used for model testing.

**Table 1:** Classification of apple leaf data set.

Type	Disease category	Training set/sheet	Validation set/sheet	Test set/sheet
0	Mosaic of apple	4275	534	534
1	Rust of apple	4525	565	565
2	Gray spot of apple	3849	481	480
3	Alternaria leaf spot of apple	3901	487	487
4	Brown spot of apple	4556	569	569

### 2.2 LALNet network model

**Figure 2:**
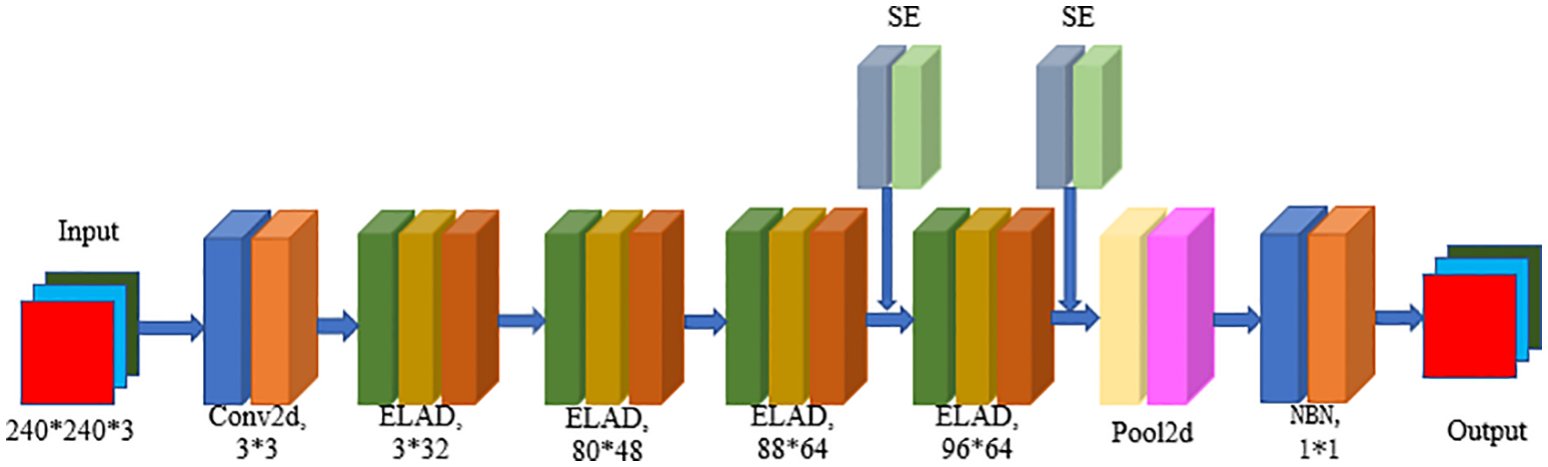
Flowchart of LALNet network structure.

The LALNet lightweight apple leaf disease identification network model was mainly constructed by referring to the typical ResNet network model and MobileNet network model structure, using depth separable modules in the network and lightweight attention modules to lighten the parameters of the network model, and using structural reparameterization in inference to improve the inference speed of the network model. The flowchart of the LALNet network was shown in **Figure 2**, which modeled the main components of the ELAD module and the SE Attention Mechanism module.

#### 2.2.1 ResNet network

In recent years, Convolutional neural nerve network (CNN) has been continuously evolving and growing, representing one of the prominent architectures in deep learning networks (Kawakura and Shibasaki, 2020; Deepan and Sudha, 2021). However, as the network depth increases, it becomes increasingly difficult to train, leading to the problem of network degradation. To address this problem, in 2015, a research team from Microsoft Research proposed ResNet (Residual Network) (He et al., 2016), a deep learning network that introduced residual connections. These connections made it easier to train deeper networks.

**Figure 3:**
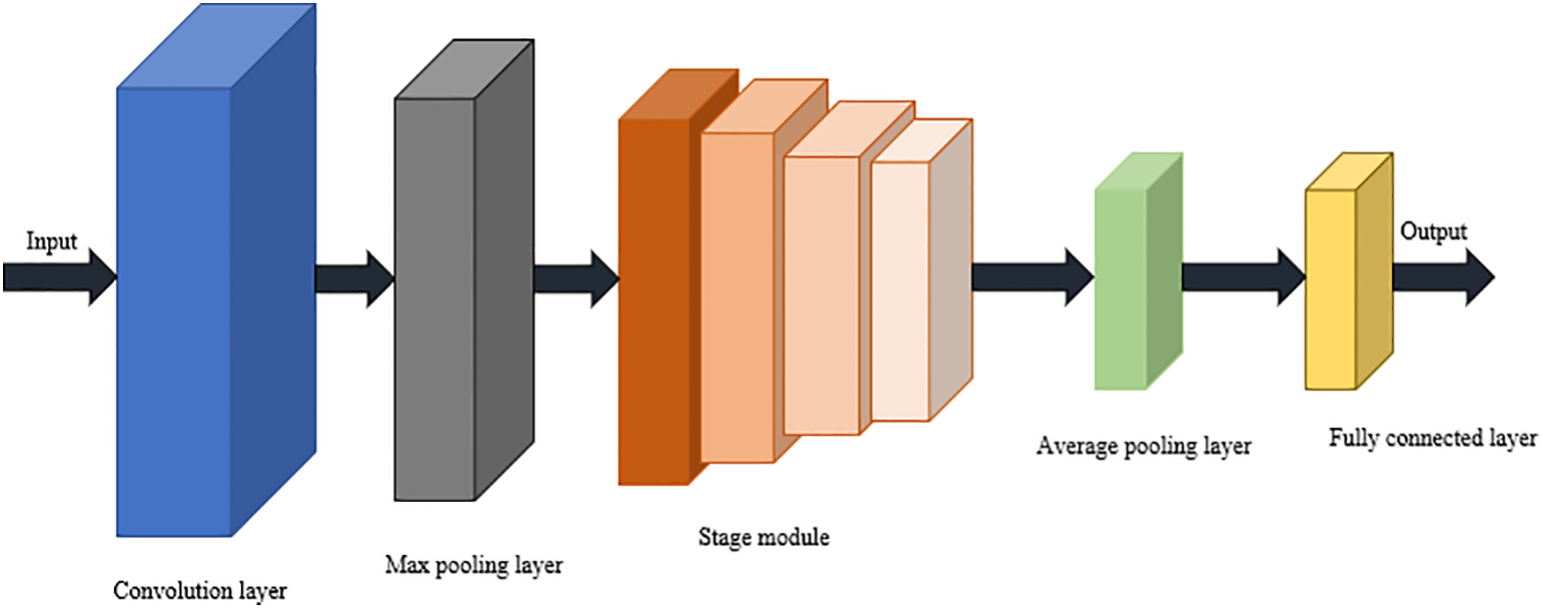
ResNet network structure diagram.

The network structure of ResNet was shown in **Figure 3**. The network mainly consists of an input layer, convolutional layers, residual modules, pooling layers, and fully connected layers. Input layer: input image data; Convolutional layer: extracts features of the image; Residual block: consists of two or more convolutional layers with residual connections; Pooling layer: reduces the dimensionality of the image; Fully connected layer: connects the outputs of all convolutional layers (Shifang et al., 2021). The network structure of ResNet consists of two main components: the residual blocks and the backbone network (Chunshan et al., 2020). Each residual block contains two or more convolutional layers and a residual connection, whose main function was to pass the residuals of the input data directly to the next residual block, which increases the mobility of the data so that the gradient can remain valid in deeper layers of the network and thus reduce the effect of gradient disappearance. ResNet constructs a deeper network by stacking more and more residual blocks to solve more complex problems.

#### 2.2.2 MobileNet network model

Lightweight network design differs from traditional neural networks by placing greater emphasis on compactness of the model structure for running networks on embedded devices. Google proposed MobileNet V1, a classical lightweight network that can be deployed on mobile (Wenjie et al., 2021), which uses deep separable convolution instead of traditional convolution to reduce the network parameters while ensuring network accuracy (Howard et al., 2019). MobileNet V2 further improves the performance of the model by adding inverse residual structure and linear units and using nonlinear activation functions in high-dimensional space based on V1. MobileNet V3 (Hu et al., 2020), based on V2, introduces lightweight attention (squeeze and excitation) (Zhou et al., 2022) modules that effectively suppress unnecessary channels, while the model uses the h-swish activation function to reduce the computational cost of applying nonlinear activation functions and achieve better parameter reduction.

**Figure 4:**
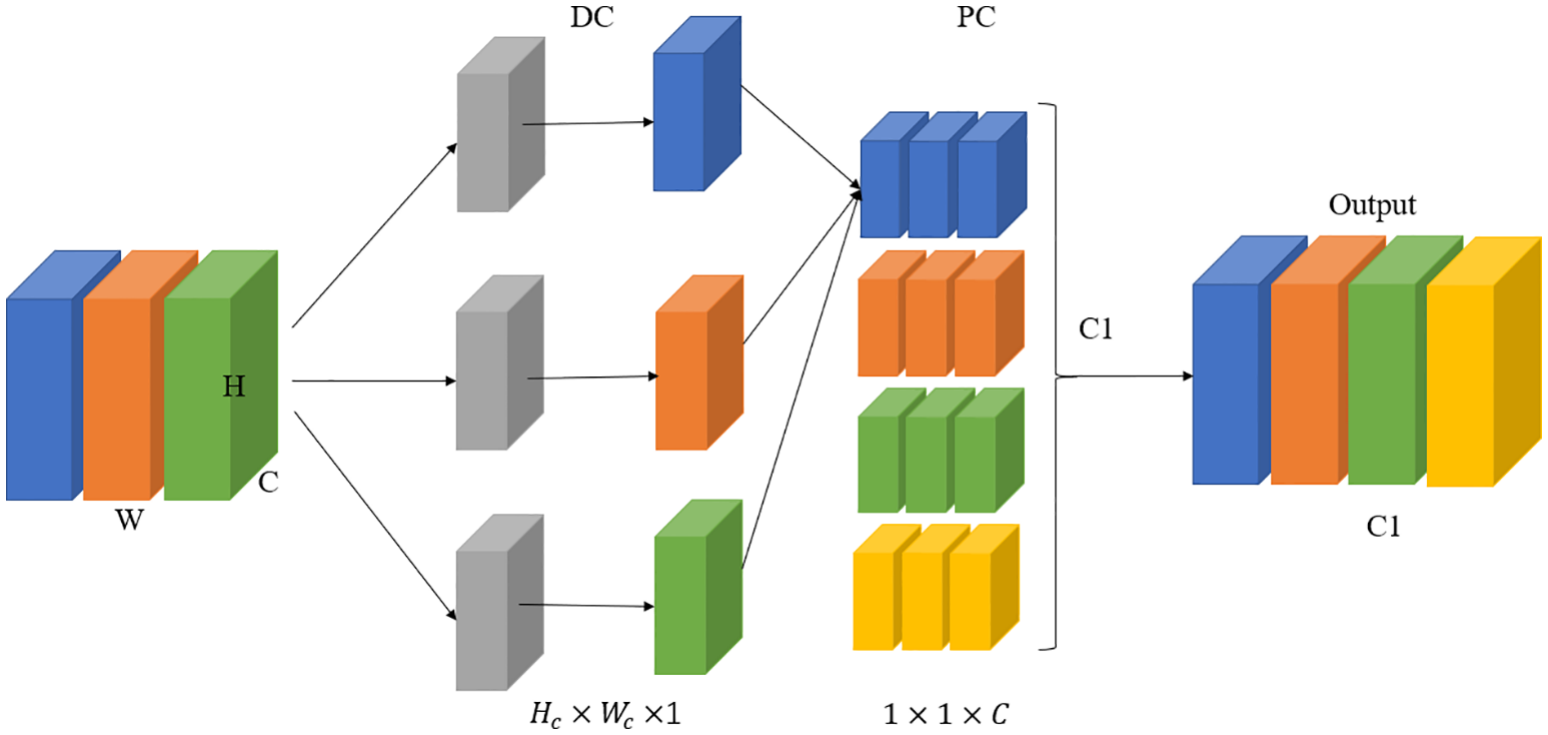
Structure of deep separable convolutional network.


Deeply separable convolution (DSC) holds the key to lightweight network design, as shown in **Figure 4**. This convolution is a decomposable convolutional structure that decomposes standard convolution into deep wise convolution, which is the process of combining features to create feature vectors of new dimensions, and Pointwise convolution, which is the process of filtering the input feature vectors. Compared to traditional convolution, deep separable convolution can reduce the parameters of the model to improve the detection speed. For example, the input feature map size for H×W, the number of input channels for M, the convolution kernel size for K×K, the number of output channels for N, and the output feature map size for OT×OT. The normal convolution computes Nc is.

(1) $$N_{c}=K\times K\times M\times N\times O_{T}\times O_{T}$$

The deeply separable convolution computation Na is.


(2) $$N_{a}=O_{T}\times O_{T}\times K\times K\times M+O_{T}\times O_{T}\times M\times N$$

The ratio of computational cost between depth wise separable convolution and regular convolution is.


(3) $$\frac{N_{a}}{N_{c}}=\frac{1}{N}+\frac{1}{k^{2}}$$

From the ratio of deeply separable convolutional to normal convolutional computation, it is shown that the reduction of deeply separable convolutional computation is related to the number of channels and the size of the convolutional kernel, with the larger the size of the convolutional kernel, the larger the computational reduction.

#### 2.2.3 Structural reparameterization

**Figure 5:**
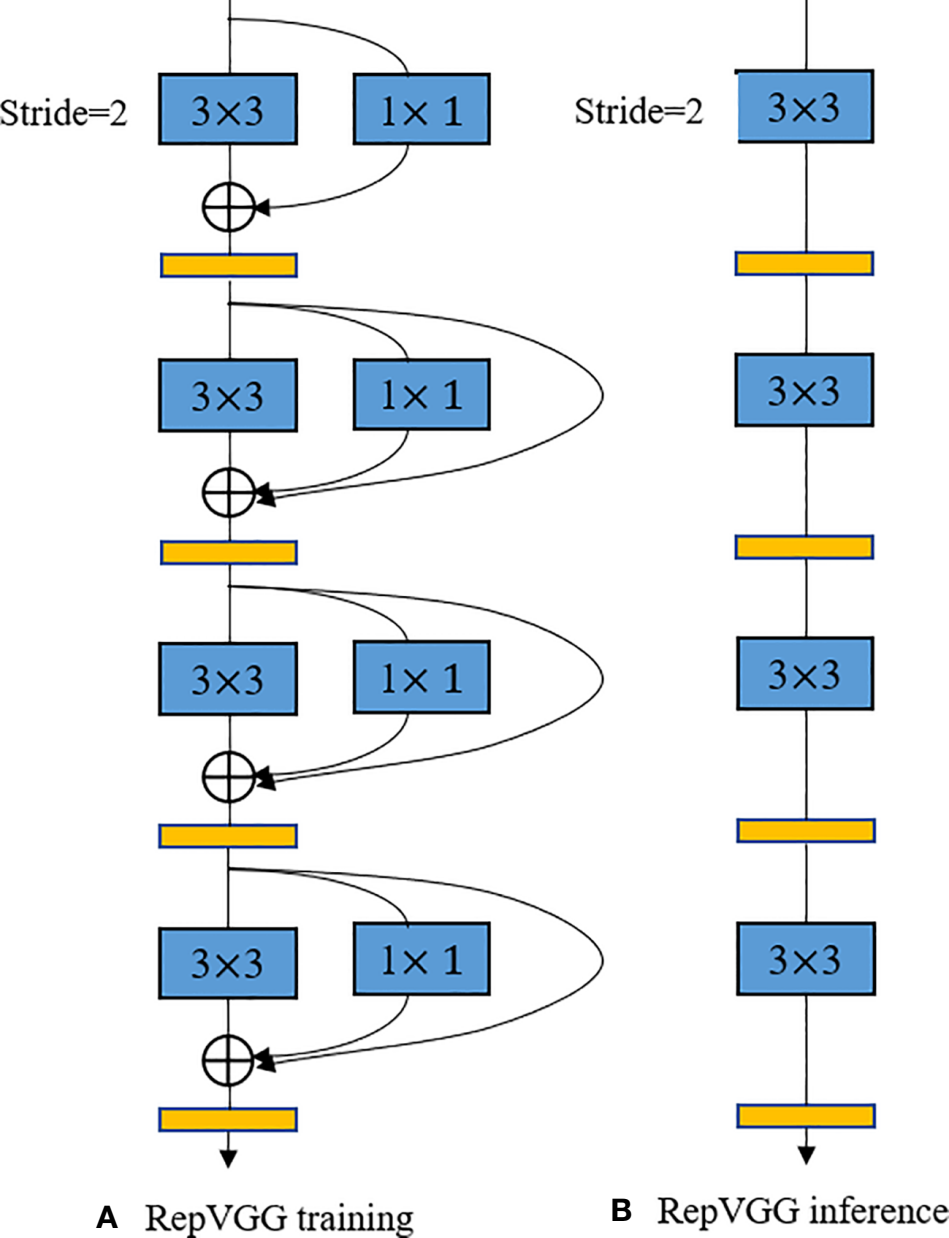
RepVGG model training and inference structure diagram.

The structural re-parameterization is a technique for optimizing neural network models (Ding et al., 2021). This technique enables efficient training and deployment of deep learning models in scenarios with limited computational resources by using constant parameter transformations to reduce the storage and computational resources of the model through simplification of the network structure. As shown in **Figure 5**, the earlier RepVGG model uses a simple architecture consisting of stacked 3*3 Conv and ReLU to achieve structural decoupling during training and inference, and uses a multi-branch structure during training, and then uses reparameterization to equivalently transform the multi-branch architecture to a VGG single-way architecture with stacked 3*3 convolutional layers after training was completed, using this structured reparameterization method to enable RepVGG to achieve ImageNet to achieve more than 80% accuracy and run several times faster (Transactions of the Chinese Society of Agricultural Engineering et al., 2021; Hu et al., 2022).

#### 2.2.4 LALNet model construction

**Figure 6:**
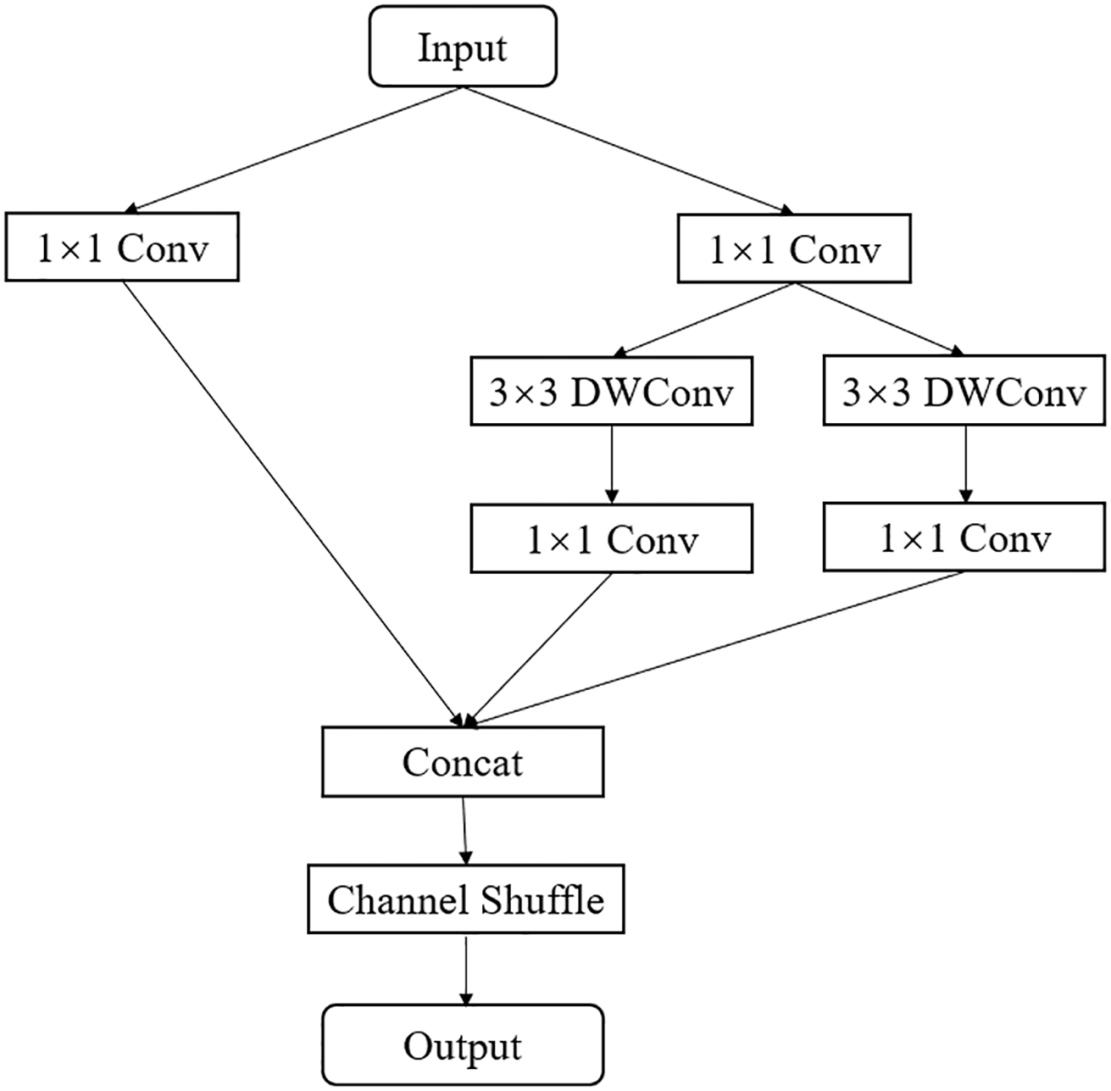
EALD module structure diagram.

Inspired by the depth-wise separable convolutions in ResNet and MobileNet, this paper proposes an efficient EALD module. The EALD module, as shown in **Figure 6**, uses a multi-branch structure and depth-wise separable modules to extract more feature information with fewer parameters and computational complexity. First, the module uses a standard 1x1 convolution kernel for dimensionality reduction, followed by different branches for feature extraction. The first and second branches use 3x3 depth separable modules to extract complex features. In the third branch, a 1x1 standard convolution is used to extract residual information and to enhance the interplay of module features. Then, the outputs of the three branches are summed and the channel number is restored using a 1x1 pointwise convolution. Finally, channel shuffling was performed to facilitate information fusion between channels, thereby improving the feature recognition capability.

The LALNet lightweight apple leaf disease classification model was stacked using the EALD module, and the network structure of the LALNet model follows in **Table 2**. First, the initialized feature extraction of three channels of the image was performed in step one using a standard convolution with a convolution kernel of 3*3, which has a step size of two and an output channel number of 16. The EALD module was used for feature extraction in steps two-five with a step size of 1. The SE attention module was added in steps four and five to increase the feature extraction capability. In step six, an adaptive averaging pooling layer was used and then a linear layer with 960 input features and 1280 output features was passed. In step seven, the output of the linear layer was passed through another batch normalization layer so that a linear layer with 1280 input features and number of output classes was applied as the final layer.

**Table 2:** network structure of LALNet model.

Step	Input	Operator	Stride	Output	Attention mechanism
1	3	Conv2d, 3×3	2	16	–
2	3	EALD,(3,32)	1	80	–
3	80	EALD, (80, 48)	1	88	–
4	88	EALD, (88, 64)	1	96	1
5	96	EALD,(96, 64)	1	96	1
6	960	Pool2d	–	–	–
7	1280	1×1, NBN		1280	–

**Figure 7:**
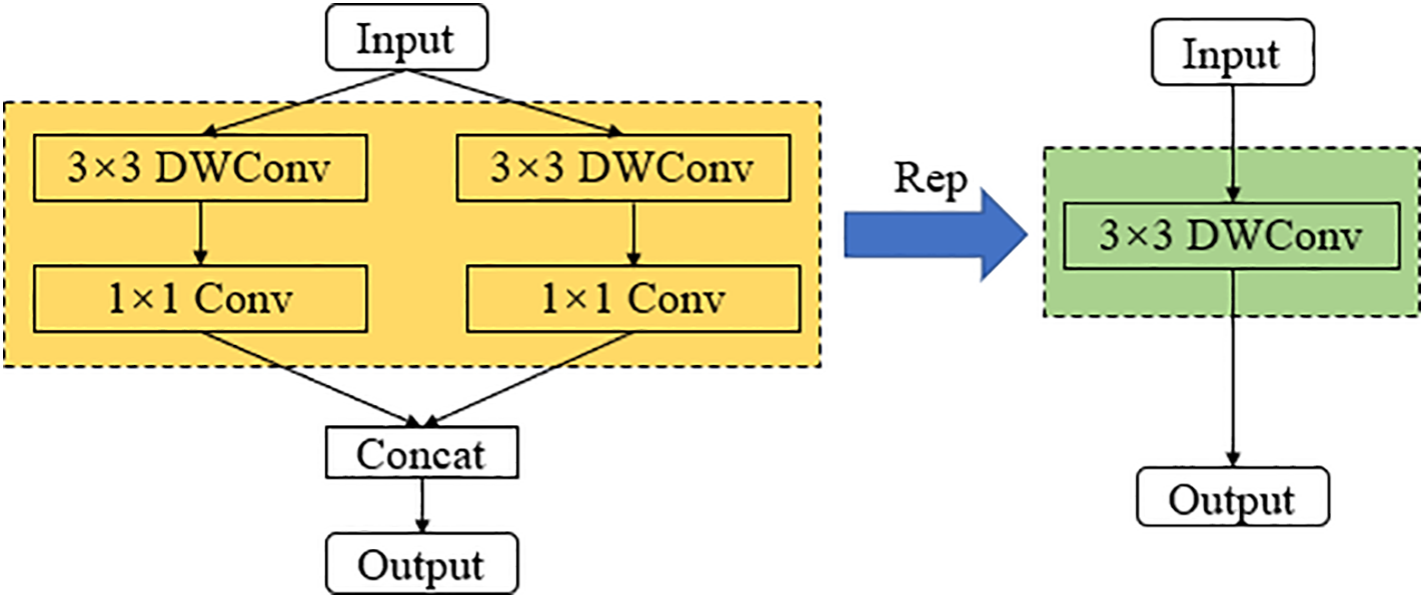
Schematic diagram of multi-branch fusion using structural re-parameters.

While a multi-branch structure reduces the number of parameters in a model, many researchers argue that having too many branches can affect the model’s runtime speed during inference. Therefore, this paper optimizes the structure of the model during recognition using a re-parameterization strategy. As shown in **Figure 7**, the convolutional layers with 3x3 depth-wise separable convolutions and their respective batch normalization (BN) layers in the first and second branches are fused. After fusion, a set of 3x3 depth-wise separable convolutional groups is used to represent the common parameters of the two branches, thereby improving the model’s recognition speed during inference.

## 3 Results and discussion

### 3.1 Experiment environment

In this study, the hardware experimental environment consisted of a Lenovo laptop (y9000p) with an Intel Pentium i5-12700H processor running at a frequency of 3.5GHz, and a GeForce GTX 3060 6G GPU. The software experimental environment involved a Windows 10 operating system, Python 3.8 as the programming language, PyTorch 1.10.0 as the machine learning library, and CUDA 10.2 as the parallel computing framework.

### 3.2 Evaluative metrics

The following metrics are commonly used when evaluating the performance of classification models:

Accuracy: This is a measure of the overall accuracy of the model’s predictions. It indicates the percentage of correct predictions made by the model across all samples.


(4) $$A=\frac{T_{P}+T_{N}}{T_{P}+T_{N}+F_{P}+F_{N}}\times 100\%$$

Precision: This is a measure of the proportion of actual positive samples for which the model predicts a positive outcome.


(5) $$P=\frac{T_{P}}{T_{P}+F_{P}}\times 100\%$$

Recall: This is a measure of the proportion of actual positive samples for which the model is predicted to be positive.


(6) $$R=\frac{T_{P}}{T_{P}+F_{N}}\times 100\%$$

F1 value: This is a combined precision and recall metric that measures the overall predictive effectiveness of the model for positive samples.


(7) $$F1=2\times \frac{P\times R}{P+R}\times 100\%$$

where: A- Accuracy; P-precision; R-recall rate;

T_P_-True positive, the number of samples correctly predicted as positive;

T_N_-True negative, the number of negative samples predicted as negative;

F_P_-False positive, the number of negative samples predicted as positive;

F_N_-False negative, the number of positive samples predicted as negative.

### 3.3 Model training, testing parameters

In training and testing the LALNet model, the parameters of the training and testing models were finally selected after several tests and trials to suit the data set and computer performance of this paper as shown in **Table 3** below, the image size of the training and testing models was 224*224, the Batch Size was 16 during training, the Batch Size was 16 during testing, the loss function was Cross entropy loss, the optimization function was Adam, the learning rate was 0.001, and the number of training rounds was 100.

**Table 3:** LALNet model training and testing parameters.

Parameter	Training	Testing
Image size	224 × 224	224 × 224
Batch Size	16	32
Loss function	Cross entropy loss	–
Optimization function	Adam	–
Learning Rate	0.001	–
Training epoch	100	–

### 3.4 Accuracy of the model training

**Figure 8:**
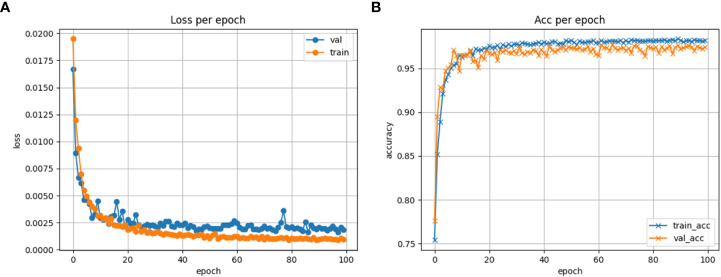
Model training monitoring graph. **(A)** Loss plot of model training. **(B)** Accuracy plot of model training.

In the training process of the model, it is common to evaluate the loss and accuracy of the training and validation sets to assess the performance of the model on the dataset (Pang et al., 2020). In this paper, both the training and validation of the model were performed using the same parameters and number of training epochs. The loss and accuracy of the model on the training and validation sets were monitored. **Figure 8A** shows the loss graph of the training and validation of the model, while **Figure 8B** shows the accuracy graph of the training and validation of the model in this paper. From the figures, it can be seen that the model initially had lower accuracy and higher loss values with significant fluctuations. However, as the training progressed, both the accuracy and loss values of the model stabilized. Therefore, the model did not experience overfitting or underfitting problems, indicating a good training performance of the model.

### 3.5 Analysis of structural re-parameterization results

In this study, structural re-parameterization was applied to the EALD module during the model inference phase, with the goal of improving the model’s runtime speed during inference. The recognition accuracy and single frame recognition speed of the model with and without structural reparameterization were evaluated on the test set (Yueming et al., 2023). The experimental results, as shown in **Table 4**, indicate that the parameter size of the model remained almost unchanged after reparameterization. Although there was a slight decrease of 1% in detection accuracy, the model’s detection speed improved by 19.03%. Therefore, this re-parameterization method demonstrates its effectiveness in improving the model running speed while maintaining the model performance.

**Table 4:** Recognition results before and after using structural heavy parameters.

Model names	Structure heavy parameters	Total accuracy of test set/%	Total precision of test set/%	Recall rate of test set/%	Test set F1 value/%	Model Parameters/MB	Single picture detection speed/ms
LALNet LALNet	Yes	95.95	95.94	98.52	96.01	6.60	8.25
	No	96.07	95.98	96.02	96.06	6.61	6.68

### 3.6 Analysis of model parameters, efficiency

**Figure 9:**
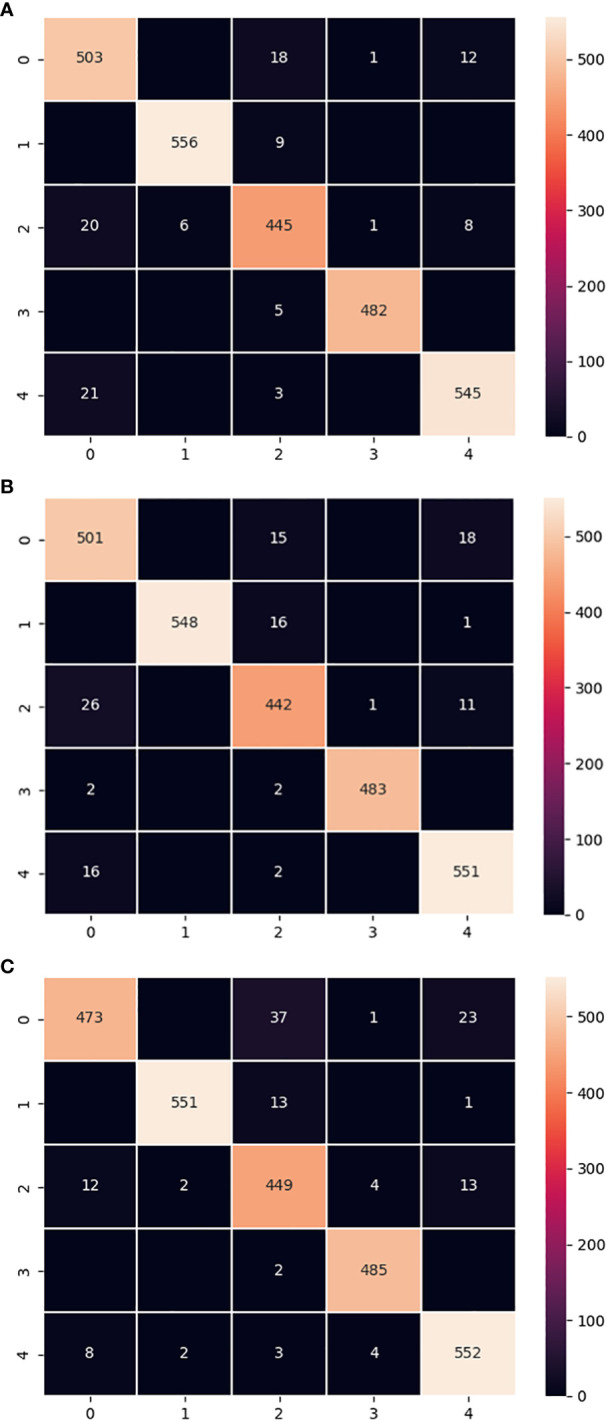
Confusion matrix for the 3 models. **(A)** LALNet model confusion matrix. **(B)** MobileNet V3-small model confusion matrix. **(C)** ShuffleNet V2 model confusion matrix.

The confusion matrix is a common tool for evaluating the performance of classifiers, which assesses the performance of the model by tracking the relationship between the actual and predicted labels of the classifier (Simonyan and Zisserman, 2014; Görtler et al., 2022). The confusion matrix of the LALNet model with MobileNet V3-small model and ShuffleNet V2 model on the test set was shown in **Figure 9**. From the confusion matrix **Figure 9A**, it can be seen that the label 0 correctly predicted images of 503, label 1’s correctly predicted images of 556, label 2’s correctly predicted images of 445, label 3’s correctly predicted images of 482, and label 4’s correctly predicted images of 545. By comparing **Figures 9A, B**, it was found that the correctly predicted images of label 4 in the MobileNet V3-small model exceeded the LALNet model, and the rest of the labels were slightly lower than the LALNet model. From the comparison of **Figures 9A, C**, it was found that the correct predicted images of label 2 in the ShuffleNet V2 model exceeded the LALNet model, and the rest of the labels were slightly lower than the LALNet model. By comparing the three confusion matrices, it was observed that each model recognized different types and numbers of confused labels, which indicated that different models had different recognition of apple leaf diseases. It was also found that the LALNet model integrated the correct label matching slightly better than the other two models, thus indicating the superior design of the LALNet model.

This paper conducts a comparative test on whether the lalnet model uses the attention mechanism. The results are shown in **Table 5**. It can be seen from **Table 5** that when the attention module is not added, the total accuracy of the LALNet model was 95.46%, the total precision was 95.79%, the total F1 was 95.43%, and the single picture detection speed is 6.68ms. When the attention module was added, the total accuracy of lalnet model was 96.07%, and the total F1 was 96.06%. The accuracy and F1 values were improved. At the same time, the single image detection speed was also slightly reduced to 7.58ms. Thus, the attention mechanism can improve the performance of the model to a certain extent, making it more accurate and robust, but it will also affect the detection speed of the model.

**Table 5:** Recognition results before and after using attention mechanism.

Name of the model	Category Tags	Attention Module	Test set accuracy/%	Test set precision/%	Test set F1/%	Single picture detection speed/ms
LALNet	0	No	94.19	94.21	93.76	7.58 ms
	1		97.52	97.50	98.31	
	2		89.58	89.58	92.18	
	3		98.36	98.38	98.97	
	4		97.01	97.11	93.96	
LALNet	0	Yes	94.19	94.22	93.32	6.68 ms
	1		98.41	98.38	98.67	
	2		92.71	92.74	92.71	
	3		98.97	98.96	99.28	
	4		98.78	98.76	96.12	

### 3.7 Comparative analysis of different models in the experimental study

**Figure 10:**
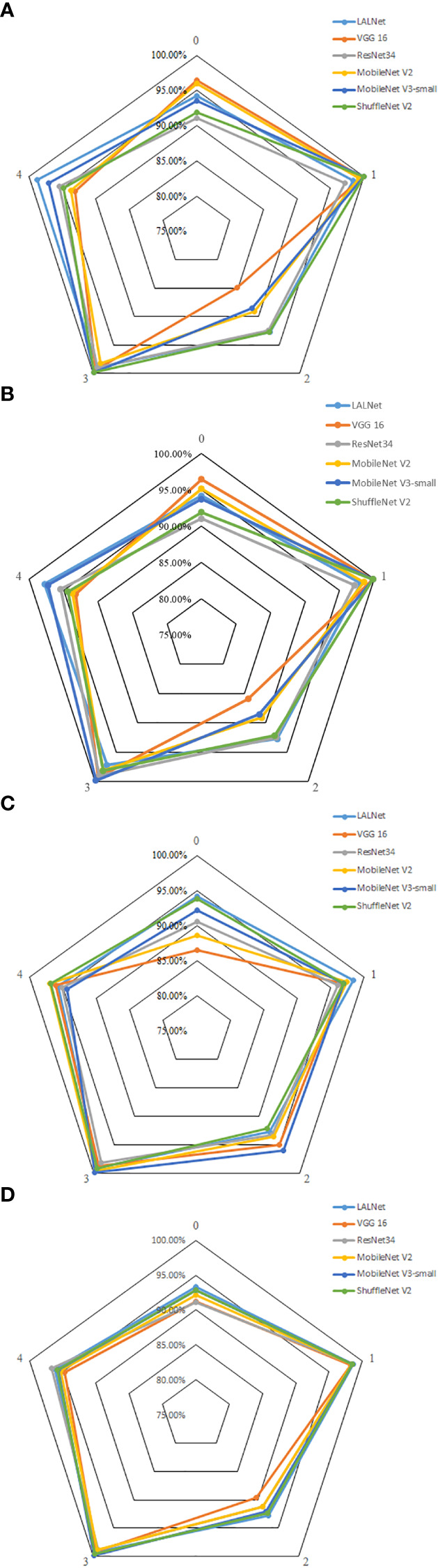
Comparison of different apple leaf disease evaluation indexes of the 6 models. **(A)** Accuracy, **(B)** Precision, **(C)** Recall rate, **(D)** F1 values.

To further validate the performance of the model in classifying different types of apple leaf categories, the model was evaluated using six different network models: LALNet, VGG16 (Ma et al., 2018), ResNet34, MobileNet V2, MobileNet V3-small, and ShuffleNet V2 (Zhong and Zhao, 2020). The experimental results on the test set are shown in **Table 6**, while the performance metrics for different leaf diseases are shown in **Figure 10**.

**Table 6:** Recognition results before and after using attention mechanism.

Name of the model	Test set accuracy/%	Test set precision/%	Test set recall rate/%	Test set F1/%	Single picture detection speed/ms
LALNet	96.07	95.98	96.05	96.06	6.68
VGG 16	94.91	94.94	94.61	94.64	10.20
ResNet34	95.03	95.00	94.42	94.45	8.46
MobileNet V2	94.93	94.91	94.51	94.64	9.68
MobileNet V3-small	95.82	95.83	95.87	95.80	8.05
ShuffleNet V2	95.25	95.28	95.26	95.20	10.23

From the data results in **Table 6**, it could be seen that the LALNet model had an overall accuracy of 96.07% on the test set, which was higher than the other six models. In addition, the total precision, the total recall and total F1 values of the LALNet model were 95.98%,96.05% and 96.06%, respectively, which were also better than the other six models. In terms of detection speed, the single image detection speed of the LALNet model was 6.68ms faster than that of the other six classical models, and the single image detection speed was 16.79% higher than that of the lightweight MobileNet V3-small model, which means that the LALNet model has better real-time detection performance in practical applications.

In the performance evaluation of the different classical models compared, VGG 16 has the lowest total test set accuracy of 94.91%, the lowest total test set F1 value of 94.64%, and the second lowest total test set recall of 94.61%. MobileNet V3-large has a test set total accuracy of 95.93% and a test set total recall of 95.87%. Comparison of the models reveals that the detection accuracy of the lightweight model MobileNet V3-small exceeds the detection accuracy of ResNet34 and VGG16 models while ensuring the detection speed, which indicates that the lightweight structure design is superior in terms of model architecture, but still lacking compared to LALNet. In comparison, it was found that the LALNet model is faster in single image detection while ensuring detection accuracy, so it is more advantageous in apple leaf disease detection application scenarios that require fast response.

As shown in **Figure 10**, the accuracy, recall and F1 values of different models varied for different leaf disease categories. The LALNet model performed consistently in terms of accuracy, fluctuating around 95% for different disease categories, with the LALNet model achieving the best accuracy for category 1 and category 4, 98.41% and 98.78%, respectively. All models performed the worst accuracy on category 2, with the VGG 16 model having a lower accuracy of only 84.92% on category 2. In terms of recall, LALNet models performed best in category 1 and category 4 on the four disease categories, while the greatest variability in performance was found among the six models in category 2, where the VGG 16 model had about 85% recall on category 2 and ShuffleNet V2 about 94% on category 2. In terms of F1 values, LALNet models had the best F1 values in categories 1 and 4, while all models had F1 values above 95% in categories 0 and 2. The comparison showed that the LALNet models performed consistently in terms of accuracy, recall and F1 values, which achieved better performance for each disease category. Compared to other models, LALNet shows superior recognition accuracy in most disease categories, further validating the reliability and effectiveness of LALNet as an excellent model for apple leaf disease recognition.

To further analyze the performance of this paper’s model in apple leaf disease detection, the LALNet model was compared and analyzed with existing state-of-the-art apple leaf disease detection methods, as shown in **Table 7**. It can be seen that the LALNet model achieves 96.07% accuracy on the self-built dataset and the Baidu AI dataset, and this paper’s model shows good performance in the disease detection task compared with other methods. In the comparison, it can be found that the detection accuracy of this paper’s model is close to or even exceeds some advanced research results, and it also can be found that the overall detection accuracy of seven models exceeds 90%, and Yinping Chen et al. achieved 97.78% on the PlantVillage dataset.However, we should also pay attention to the limitations of different methods due to the experimental environments in which the hardware devices different and the datasets used are also very different, which will affect the test results, especially the detection speed of the model.

**Table 7:** Comparison results of advanced apple leaf classification models.

Reference	Data sets	Disease type	Model	Accuracy(%)
Yong Zhong et al. (Zhong and Zhao, 2020)	AI challenger plant disease recognition	Healthy apple, General apple scab, Serious apple scab, Apple gray spot, General cedar apple rust, Serious cedar apple rust	Densenet-121	93.71
Yuxi Gao et al. (2023)	Kaggle dataset + Self built dataset	Healthy, Brown spot, Alternaria leaf spot, Mosaic, Powdery mildew, Rust	BAM-Net	95.64
Xiaofei Chao et al. (2021)	Self built dataset	Alternaria leaf spot, Rust disea, Brown spot disease, Gray spot disease, Mosaic disease, Healthy leaves	SE_miniXception	97.01
Cong Xu et al. (Cong et al., 2022)	Self built dataset	Mosaic, Brown spot, Rust, Gray spot, Spotted leaf litter	DCCapsNet	93.16
Yinping Chen et al. (Yiping et al., 2023)	Plant village	Healthy, Scab, Cedar rust	Inproved CycleGAN	97.78
Chongke Bi et al. (2022)	Self built dataset	Alternaria leaf, Spot, Rust	MobileNet v3	73.50
Helong Yu et al. (2022)	Kaggle dataset	Powdery mildew, Alternaria leaf spot, Scab, Rust, Alternaria leaf spot and rust, Healthy	MSOResNet	95.7
Proposed method et al.	Self built dataset+ Baidu AI dataset	Mosaic, Rust, Gray spot, Alternaria leaf spot, Brown spot	LALNet	96.07

## 4 Conclusion and limitations

In this paper, it proposed a fast apple leaf disease detection model LALNet. Firstly, an efficient leaf detection stacking EALD module was designed using multi-branch structure and depth separable modules, which can obtain more accurate identification information with less parameters and computation. Further, the EALD module was used in the LALNet model to stack four layers and add the SE module in the last layer of the model to improve the attention of the network model to focus on important features. Finally, the structural reparameterization technique was used to combine the outputs of two layers of deeply separable convolutions in the branch to improve the speed of the model during the inference phase. The proposed fast apple leaf disease detection model has an overall accuracy of 96.07% in the test set, precision of 95.98%, and F1 score of 96.06%, a model size of 6.61 MB, and a detection speed of 6.68 ms for a single image, thus the model meets the detection accuracy while ensuring its operation speed and is suitable for use on embedded devices.

However, it is important to acknowledge the limitations of this study in order to provide readers with a comprehensive assessment. First, the dataset used in this research has limitations in terms of data collection methods, sample size, and range of disease types covered, which may affect the generalizability of the model. Second, the performance of the model in real-world applications may be affected by factors such as lighting variations, different capture angles, and variations in leaf quality, which may affect its detection performance. Finally, while the focus of this study was on common apple leaf diseases, it does not cover all possible disease types that may be present in practical cultivation. Future research should consider collecting more diverse and comprehensive datasets and further optimizing the model to improve its accuracy and robustness.

In future work, we aim to further improve the performance of the LALNet model by addressing the aforementioned limitations. In addition, we plan to use the model in an intelligent tracked apple spraying robot to achieve precision spraying and reduce pesticide use.

## Data availability statement

The original contributions presented in the study are included in the article/supplementary material. Further inquiries can be directed to the corresponding authors.

## Author contributions

RH prepared materials. ZY was responsible for the experiment design. GA and RL performed the program development. GA, and HX analyzed the data, and SY were responsible for writing the manuscript. SY and RL contributed to reviewing the manuscript. All authors contributed to the article and approved the submitted version.
